# High-Temperature Surface Energy and Interfacial Energy of the Molten FLiNaK–Highly Oriented Pyrolytic Graphite (HOPG) System from Selected Contact Angle Measurement

**DOI:** 10.1021/acs.iecr.6c02718

**Published:** 2026-09-18

**Authors:** Jisue M. Braatz, Gang Seob Jung, Nidia C. Gallego

**Affiliations:** † Radioisotope Science Technology Division, 6146 Oak Ridge National Laboratory, 1 Bethel Vally Rd, Oak Ridge, Tennessee 37830, United States; ‡ Computational Sciences and Engineering Division, Oak Ridge National Laboratory, Oak Ridge, Tennessee 37830, United States; § Chemical Science Division, Oak Ridge National Laboratory, 1 Bethel Vally Rd, Oak Ridge, Tennessee 37830, United States

## Abstract

Accurate high-temperature surface and interfacial energies are essential for understanding molten salt interactions with nuclear graphite. This study examined how initial pellet geometry and temperature affect the wetting of FLiNaK on highly oriented pyrolytic graphite (HOPG). Sessile-drop experiment using 1.5 m chunk and 3 mm pellet showed that precursor geometry influences transient contact angles and the time required to reach a common long-time value. Capillary forces dominated, while minor gravity effects for 3 mm pellets could not be excluded. Observed variations were mainly attributed to droplet shape evolution and contact-line pinning. The Neumann equation of state was used to estimate HOPG surface energy, yielding approximately 145 mN/m at 500 °C and 120 mN/m at 800 °C. Solid–liquid interfacial energies were consistently 30–40 mN/m below solid–gas values, indicating partial wetting dominated by physical interactions. These findings establish a reproducible method and show that contact angle convergence must be demonstrated before interfacial energy analysis.

## Graphical abstract

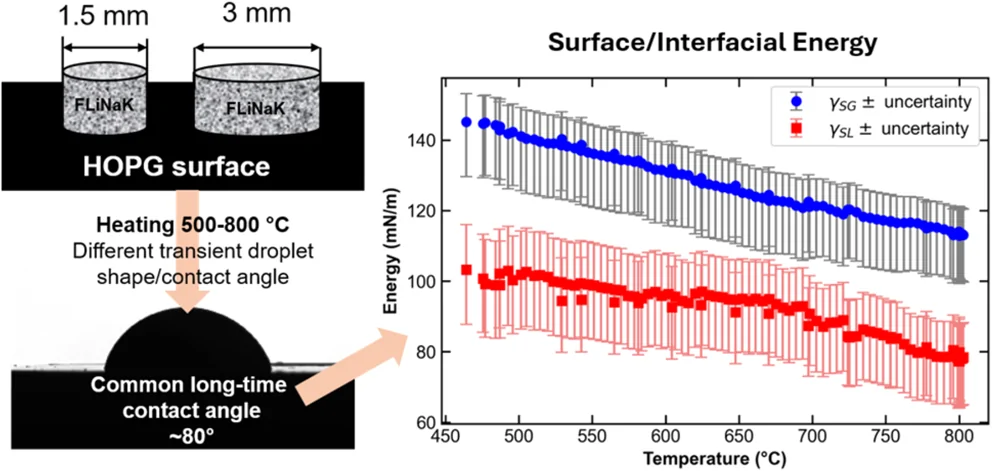

## Introduction

Understanding the interfacial properties of molten salt with carbon-based materials is critical to the design and long-term operation of molten salt reactors (MSRs).^1,2^ Graphite materials serve as moderators, reflectors, and structural materials in fluoride salt–cooled systems, and their performance vitally depends on resistance to salt infiltration and wetting-driven degradation.^2–4^ Determining liquid wettability and the corresponding solid surface energy of materials under high-temperature conditions is essential for predicting and managing interactions between molten salts and graphite materials in MSRs. Accurate determination of surface and interfacial energies is particularly important for interpreting wetting phenomena and predicting material behavior when in contact with molten salts.

A solid’s surface energy is the excess free energy per unit area associated with creating a surface compared with the bulk.^5^ In solid–liquid systems, the balance between the solid–gas (γ_SG_), solid–liquid (γ_SL_), and liquid–gas (γ_LG_) interfacial energies defines the equilibrium contact angle (θ) according to Young’s eq (Figure 1).

**Figure 1:**
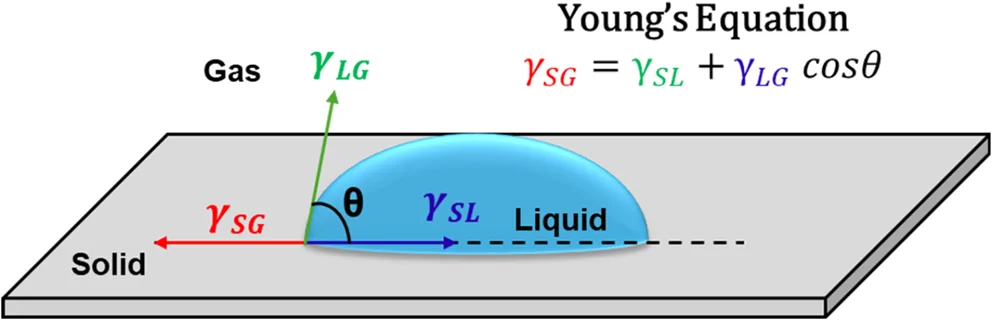
Young’s equation and construction of the force balance at a three-phase contact line between a droplet, gas, and solid surface.

For graphite materials, these interfacial parameters control key physical processes such as salt infiltration into pores, capillary transport along microstructural features,^6,7^ and the stability of the graphite–salt interface under irradiation and thermal cycling.^8^ Without quantitative values for surface energy (γ_SG_) or interfacial energy (γ_SL_), using contact angle measurements alone provides information only about wettability to determine whether the material is hydrophobic or hydrophilic. By converting contact angle measurements into apparent surface and interfacial energy values, it becomes possible to compare different graphite materials on a consistent thermodynamic basis, evaluate the influence of crystallite orientation or impurities, and benchmark computational predictions of molten salt–graphite interactions.

Because surface energy (γ_SG_) and interfacial energy (γ_SL_) vary with temperature, high-temperature measurements are essential for assessing graphite’s long-term performance in the reactor environment. As shown in recent studies on molten fluoride salt infiltration in nuclear graphite, the combination of the surface energy of a solid (γ_SG_), the liquid surface tension (γ_LG_), and pore size distribution controls the extent of salt penetration, which directly affects mechanical integrity and radionuclide retention.^7,9–11^ Therefore, the quantitative interfacial energy (γ_SL_) calculation is a matter of fundamental interest and a prerequisite for predictive modeling and safe design of graphite components in MSRs.

The sessile drop method measures the contact angle of a liquid droplet resting on a solid substrate. It provides a straightforward method to evaluate wettability and interfacial energies. Applying the sessile drop method at high temperatures presents several challenges, including chemical reactivity at the drop–substrate interface, vaporization of molten components, and optical distortion in the furnace environment. Additionally, when studying high-temperature systems in which the solid sample melts into a liquid droplet instead of directly dosing liquid onto the surface, controlling the initial shape of the solid is essential because the melt inherits the geometry of the initial solid. Irregular initial shapes can distort the droplet profile, introduce errors in the measured contact angle, and compromise the reliability of surface or interfacial energy analysis. The initial geometry of a solid salt pellet, including its height, diameter, and surface curvature, can transiently pin the triple line, defined as the boundary where the solid, liquid, and gas phases meet, and yield apparent contact angles that do not represent the equilibrium wetting state.^12,13^ Only after complete melting and capillary relaxation does the droplet attain a shape consistent with the interfacial balance dictated by Young’s equation.

Contact-angle hysteresis represents the range of metastable apparent contact angles sustained by pinning of the three-phase contact line. In a conventional measurement, the advancing contact angle, θ_A_, is obtained by increasing droplet volume until the contact line advances, whereas the receding contact angle, θ_R_, is obtained by decreasing the volume until the contact line recedes. The difference between these limiting angles reflects surface heterogeneity, roughness, chemical variation, and contact-line pinning. In the present high-temperature configuration, molten salt could not be added to or withdrawn from the droplet in situ. Therefore, formal advancing and receding contact angles were not measured. Instead, thermal cycling was used to examine path dependence, contact-line pinning and depinning, and recovery of the stabilized apparent contact-angle state.^5,12,13^

Conventional practice often assumes that equilibrium contact angles are independent of initial droplet geometry and that extracted values directly reflect intrinsic solid–liquid energetics.^12,13^ Although this assumption holds for many ambient-temperature systems due to the easier dosing method for a room-temperature liquid, its validity for molten salt, which has high surface tension (∼200 mN/m for FLiNaK [eutectic mixture of LiF–NaF–KF] near 600 °C) and abrupt melting transitions, remains uncertain. Addressing this gap requires experimental protocols that explicitly account for the solid-to-liquid shape transition and verify the reproducibility of the molten equilibrium geometry. Therefore, careful control of initial pellet geometry is essential to avoid systematic errors when extracting interfacial energies from high-temperature sessile drop data.^12–14^

Highly oriented pyrolytic graphite (HOPG) was selected as a model substrate in this study to probe graphite–salt interactions because it provides a relatively atomically smooth, chemically homogeneous basal plane surface that minimizes complications from surface roughness, grain boundaries, or porosity, which are inherent to nuclear-grade graphite.^14,15^ This makes HOPG an ideal model material for isolating fundamental wetting and interfacial phenomena.^9^ Fresh cleavage of HOPG exposes predominantly the graphite basal plane. Accordingly, the γ_SG_ values reported here should be interpreted as effective surface free energies of this basal-plane-dominated surface rather than of graphite in general. Graphitic surfaces are anisotropic, and edge or prismatic planes, grain boundaries, defects and chemically modified sites can exhibit different surface energetics and contact-line pinning behavior. Reactor-grade nuclear graphite is polycrystalline and porous, with distributions of crystallite orientation, roughness, defects and connected pores that can alter apparent contact angles and promote capillary infiltration. Thus, the present HOPG measurements provide a model basal-plane benchmark, not a direct surrogate for the wetting response of nuclear graphite and develop a protocol for measuring the contact angle of molten salt at high temperature.

FLiNaK (eutectic mixture of LiF–NaF–KF; 46.5 LiF, 11.5 NaF, and 42 KF mol %) is a prototypical molten fluoride salt with established thermophysical properties and widespread relevance in MSR technology.^1,2,15^ Together, FLiNaK–HOPG offers an ideal experimental platform for investigating wetting and interfacial energetics under high-temperature conditions. Established high-temperature sessile-drop practice emphasizes control of atmosphere and impurities, reproducible substrate preparation, assessment of droplet size relative to capillary and gravitational scales, time-resolved imaging, and sufficient isothermal holding to distinguish transient spreading from a stable apparent state.^5,12,16–19^ The present expeirments follow these principles through purified FLiNaK handling in a low-moisture glovebox, freshly cleaved HOPG, ultrapurity–purity argon with an impurity trap, one-second bilateral angle acquisition, Bond-number and capillary-length analysis and extended isothermal holds.

In this study, we investigated the wetting behavior of FLiNaK on HOPG from 500 to 800 °C with a focus on three unresolved questions: (1) Does droplet geometry influence the apparent equilibrium contact angle? (2) How does the geometric size of a solid FLiNaK pellet influence the initial apparent contact angle? (3) Can the solid surface and interfacial energies be quantitatively determined from single-liquid contact angle data? We showed that molten FLiNaK droplets retain a measurable dependence on the initial geometry of the solid pellet, underscoring the need for careful initial solid pellet preparation in high-temperature sessile drop experiments. We then applied the Neumann equation of state (EOS) method^20,21^ to invert contact angle and surface tension data to calculate the apparent surface energy (γ_SG_) and associated interfacial energy (γ_SL_) of HOPG. Sensitivity analysis confirmed that the extracted values are reliable despite the empirical nature of the EOS interaction parameter. The results provide a reproducible methodology for high-temperature surface and interfacial energy measurements and establish a baseline for interpreting the wetting of nuclear-grade graphite materials in MSR environments.

## Experimental Section

### Materials

HOPG, grade SPI-1 (commonly referred to as ZYA grade), was selected as the solid substrate for this study. It was purchased from SPI Supplies and had dimensions of 10 × 10 × 2 mm (width × length × thickness). SPI-1 HOPG was chosen for its superior crystallographic order, smooth basal surfaces, and low mosaic spread.

The FLiNaK eutectic salt mixture used in the study was prepared in the laboratory and purified by the fluorination procedure; additional information, including data on impurities, can be found in the references.^22^ After purification, the molten FLiNaK salt was broken into chunks, ground into a fine powder, and pressed into pellets of various shapes. FLiNaK chunks approximately 1.5 mm were also collected separately. All handling of FLiNaK salt was conducted in a glovebox with low water and oxygen contents (<0.1 ppm).

Three distinct two-dimensional shapes were prepared for experimentation: a flat-sided rectangular shape and trapezoidal shapes with either short-side or long-side contact with the HOPG substrate. The trapezoidal pellet was pressed using a hand press with a 3 mm die (PIKE Technologies), whereas the flat-sided pellet was pressed using a hydraulic press equipped with a 3 mm die set (MSE Supplies). The weight of each FLiNaK pellet, measured inside the glovebox, was around 10 mg (±1 mg). Each weight was double-checked before measuring the contact angle.

### Contact Angle Measurements

Contact angle measurements were performed using a Linseis L74 optical dilatometer, which is a noncontact instrument that enables high-resolution optical imaging and real-time monitoring of sample geometry during thermal processing. This instrument is equipped with a high-resolution CCD camera that allows for precise observation of materials at elevated temperatures (see schematic in Figure 2). Specifically, the dilatometer was used to study the wetting behavior of the molten FLiNaK salt sessile drop on the HOPG surfaces. The system operates under an inert gas (argon) atmosphere to prevent oxidation or contamination of the sample during heating. The image and contact angle were recorded every second, and contact angles were recorded from both the left and right sides of the droplet. Each pellet configuration was evaluated in two independent heating experiments (*n* = 2 independent runs per configuration). For every run, contact angles were recorded separately from the left and right sides of the same droplet. These two values were treated paired with measurements that reflect droplet asymmetry and image-analysis variability, rather than as independent experimental replicates. For the reproducibility assessment, the left- and right-side angles were first averaged at each time point, and the resulting mean apparent contact angle was averaged over the stabilized interval of the experiment. The relative percent difference between the stabilized mean values obtained from the two independent runs was up to 3%.

**Figure 2:**
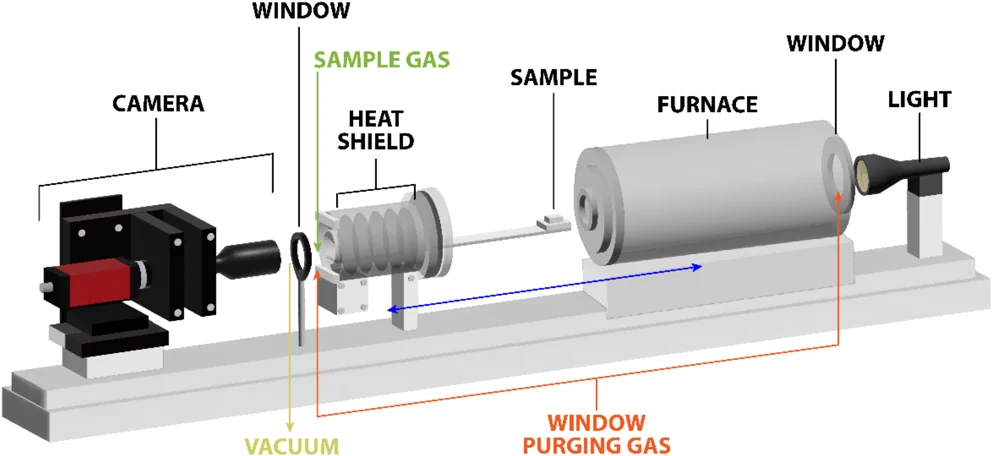
Linseis L74 optical dilatometer diagram.

The HOPG substrates were stored in a vacuum desiccator and peeled before being placed in the dilatometer sample holder to ensure surface consistency and to use a freshly cleaved surface for the contact angle measurement. Preshaped 3 mm diameter salt pellets and 1.5 mm salt chunks were prepared in the glovebox with low water and oxygen contents (<0.1 ppm).

The FLiNaK–HOPG salt pellets were placed on the sample pad inside of the dilatometer furnace. The furnace enclosure was then repeatedly evacuated and backfilled with argon to ensure a fully inert atmosphere. Ultrahigh-purity argon gas was used, and an in-line impurity trap (Drierite gas purifier, WA Hammond Drierite Company) was installed before the dilatometer gas inlet to ensure any moisture present in the argon gas was captured.

For the contact angle measurements, two different heating profiles were used in this study to understand the effect of the initial shape of the solid pellet.

1. Ramp-to-800 °C Isotherm Profile: In the first set of experiments, the salt was heated from room temperature to 400 °C, which is a temperature below its melting point (approximately 454 °C), at a relatively high rate of 10 °C/min to reduce experimental time. As the temperature approached the melting region, the heating rate decreased to improve the resolution of thermal changes. Specifically, after reaching 400 °C, the temperature was held for approximately 30 min to ensure thermal equilibrium, then increased from 400 to 800 °C at a slower rate of 2 °C/min to more accurately capture gradual transitions. Finally, the sample was maintained at 800 °C for 30 min before initiating cooling. An additional experiment was conducted to examine heating–cooling path dependence. After melting temperature of FLiNaK, the FLiNaK-HOPG specimen was cycled between approximately 500–700 °C at a nominal rate of 2 °C/min, following a 500 → 700 → 500 → 700 °C sequence, and was then cooled.
2. Stepped Isothermal Profile: For the second heating profile, the sample was heated to 550 °C at a rate of 10 °C/min and held at this temperature for 3 h. The temperature was then increased from 550 to 650 °C using a slower ramp rate of 2 °C/min, followed by an additional hold for 3 h at 650 °C. Finally, the temperature rose from 650 to 750 °C at 2 °C/min and was kept at 750 °C for 3 h.

### Molecular Dynamics

Molecular dynamics (MD) simulations were performed using the LAMMPS package.^23^ The simulations were conducted to evaluate the surface energy of graphite and its temperature dependence based on slab–bulk energy differences. Because surface energy calculations are sensitive to the in-plane surface area, sufficiently large simulation cells were constructed (Figure S2) to minimize finite-size effects associated with lateral fluctuations.

To ensure structural stability while maintaining computational efficiency, smaller prototype systems were constructed and equilibrated before generating full-scale models. Both bulk and slab configurations were then equilibrated for 0.5 ns under the NPT ensemble.

For the bulk system, fully anisotropic cell relaxation was applied at 300 K and 1 atm. In contrast, for the slab system, only the in-plane directions (*x* and *y*) were allowed to relax under the same thermodynamic conditions. A vacuum region exceeding 5 nm was introduced along the *z*-direction to prevent interactions between periodic images. Along the *c*-axis, the slab consisted of 14 graphene layers, corresponding to an overall thickness of approximately 5 nm.

The equilibrated structures were used as initial configurations for simulations at each target temperature. Before production runs, the systems were replicated in the *x* and *y* directions to obtain sufficiently large lateral dimensions and reduce finite-size effects associated with thermally excited out-of-plane fluctuations. Because long-wavelength bending modes introduce size-dependent behavior in graphene-based systems,^24,25^ the lateral dimensions were increased to approximately 40 × 40 nm.

For each target temperature, additional equilibration was performed while monitoring fluctuations in total energy and surface area to ensure statistical stability. When necessary, an additional 0.5 ns of relaxation was applied until steady-state behavior was achieved. The relative standard deviation (standard deviation divided by the mean) of the surface area was reduced to approximately 3 × 10^–4^, and that of the total energy reached 2.0 × 10^–5^, indicating well-converged thermodynamic sampling.

Subsequently, 0.5 ns production simulations were carried out for each configuration. The surface energy was computed using time-averaged total energies and surface areas, and the corresponding standard deviations were used to estimate statistical uncertainty (error bars).

## Results and Discussion

### High-Temperature Contact Angle Measurements for FLiNaK–HOPG

To examine whether the initial pellet geometry influences the apparent contact angle of molten FLiNaK on HOPG, two pellet geometries and three configurations, depending on the placement of the trapezoidal pellet, were prepared using a hand-pelletizer: (1) a thin pellet with a trapezoidal cross section from the side view (narrow side contacting the substrate), (2) trapezoidal pellets with the wide side contacting the substrate, and (3) pellets with a straight, vertical edge, giving an initial contact angle close to 90°. Experiments were conducted with the ramp-to-800 °C profile; the results are summarized in Figure 3.

**Figure 3:**
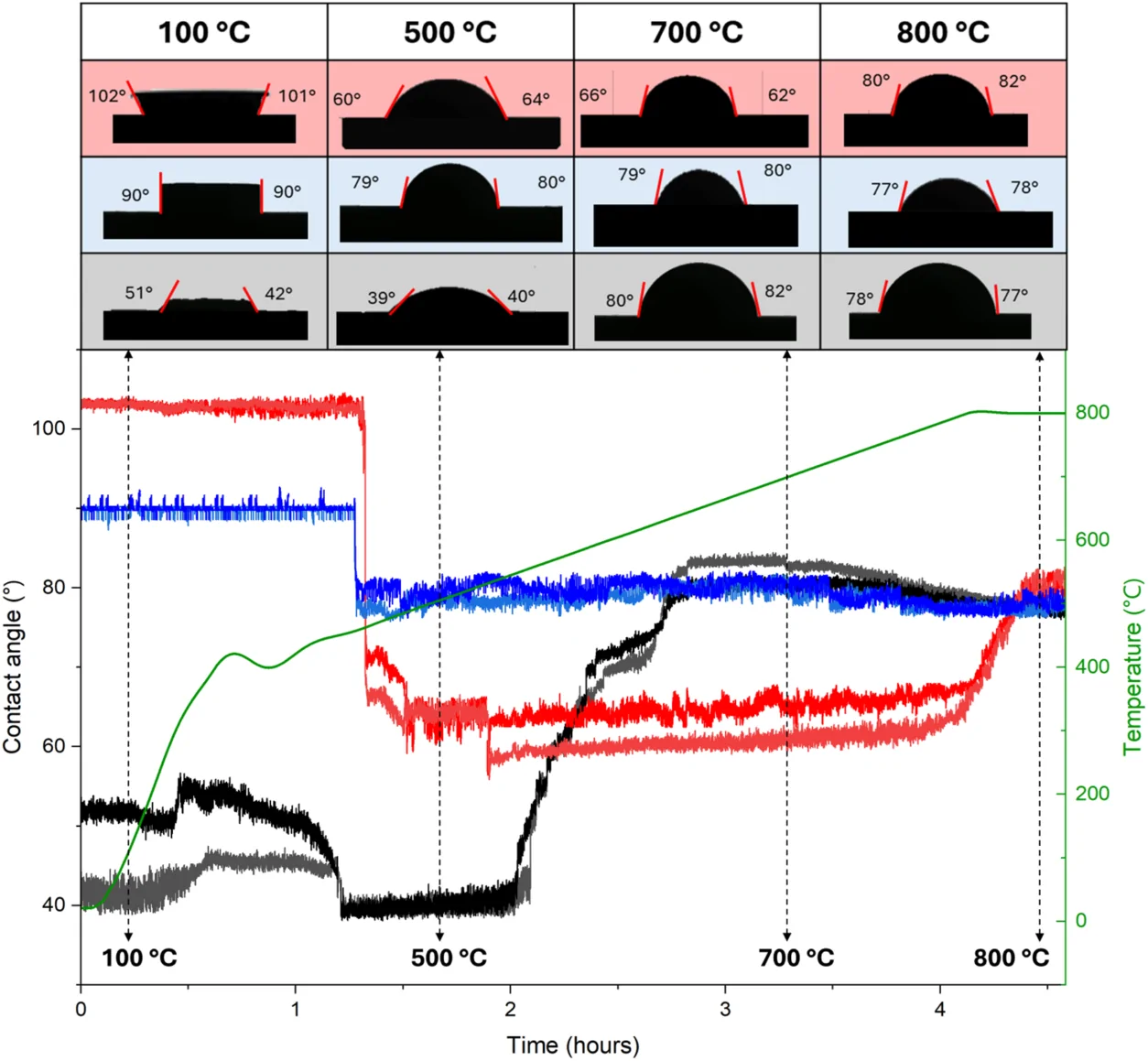
Apparent contact angle of FLiNaK on HOPG as a function of temperature. Darker shades represent the left-side contact angle measurements, while lighter shades represent the right-side contact angle measurements. Three 3 mm pellet configurations were investigated: (blue) straight-edge pellet, (black) angled pellet with longer side facing down, and (red) shorter side facing down on the HOPG. The green curve indicates the temperature profile during heating. Vertical dashed lines mark the temperatures corresponding to the representative droplet images shown above the graph (100, 500, 700, and 800 °C). The top panel displays representative contact angle images at these temperatures, with each row corresponding to the pellet configuration color used in the graph. Each plotted data set represents one representative heating experiment and is not an average of replicate runs.

When the pellet contacted the HOPG with its narrow trapezoid edge, the initial solid-state contact angle was approximately 102°. Upon melting at 454 °C, the contact angle rapidly decreased to 71° (left side) and 66° (right side). The contact angle gradually dropped to approximately 60° as the temperature increased to 514 °C and remained fairly constant until the temperature reached about 780 °C, above which the contact angle increased again to approximately 80°. In contrast, when the wide trapezoid face touched the substrate, the contact angle stabilized near 40° after melting but subsequently increased sharply above 545 °C, reaching approximately 80° after heating at 634 °C. Finally, when the pellet was prepared with a straight, vertical edge (90° side contact), the contact angle remained relatively stable around 80° across the full temperature range and during the 30 min hold at 800 °C, decreasing only slightly from 80° to 77°. All configurations converge toward a similar apparent contact angle despite dramatically different initial geometries. Figure 3 presents one representative time-resolved experiment for each pellet configuration and is not an average of the replicate runs. Each configuration was tested in two independent experiments. Within each run, the left-and right-side contact angles provide paired measurements of the same droplet and indicate the degree of profile asymmetry. Reproducibility was assessed using the stabilized apparent contact angle calculated by first averaging the left- and right-side values and then averaging over the selected stabilization interval. The stabilized values from the duplicate experiments agreed up to 3%, demonstrating that the convergence toward the approximately 80° apparent contact-angle state was reproducible within the experimental resolution. Because only two independent runs were conducted for each condition, the individual values and relative percent difference are reported rather than treating the data as a large statistical population. Comparison table of two runs is listed in Table S1.

These results suggest that when the initial solid contact angle is not perpendicular on the surface (≠90°), the system undergoes a time-dependent transition toward an intermediate contact angle. To explore whether this transition is governed more strongly by time or temperature, an additional experiment was performed with pellets exhibiting very low initial contact angles (<50°), made with the hand pelletizer. The sample was heated using a stepped isotherm (550, 650, and 750 °C), with each temperature held for 3 h. As shown in Figure 4, although melting was sluggish at 550 °C due to limited heat transfer from a faster heating rate than the earlier experiment, the contact angle eventually increased toward approximately 80°, where it stabilized during each subsequent hold.

**Figure 4:**
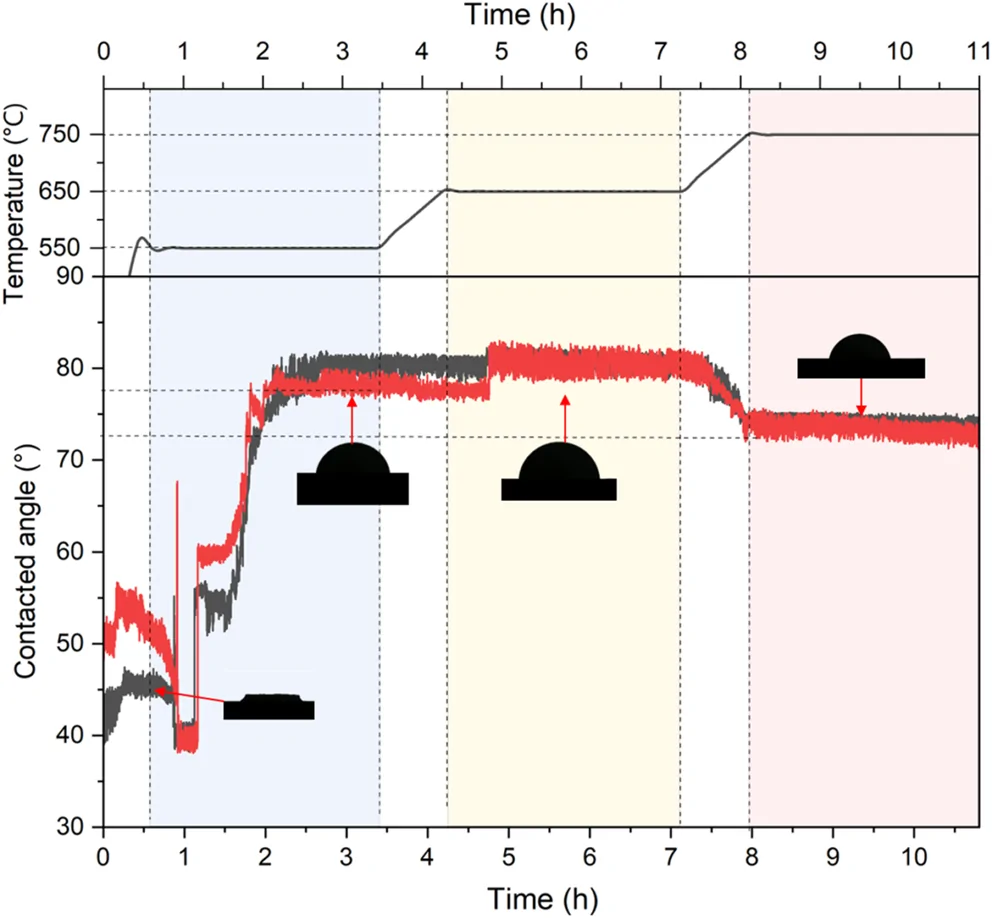
Contact angle (black indicates left-side contact angle and red indicates right-side contact angle of the droplet) evolution of FLiNaK on HOPG for pellet. Figure shows initially low contact angles (<50°) during stepwise heating to 550, 650, and 750 °C, with 3 h holds at each temperature. The contact angle initially increased rapidly after melting and then stabilized near approximately 80° during each hold regardless of temperature.

The three initial configurations evolved toward a common apparent contact angle of approximately 80° over the experiment time scale. This convergence is consistent with an equilibrium or near-equilibrium wetting state under experimental conditions. However, thermodynamic equilibrium was not independently established by reversible advancing/receding measurements or by a controlled heating–cooling cycle. Accordingly, approximately 80° is referred to hereafter as the stabilized or long-time apparent contact angle.

To evaluate the heating–cooling path dependence, an additional specimen was cycled between approximately 500 and 700 °C (Figure S1). During the first heating branch, the mean apparent contact angle remained 62.8 ± 0.8°, while the reported droplet width remained nearly constant at 3.024 ± 0.007 mm, consistent with persistence of a pinned low-angle configuration. During the first cooling branch, however, the apparent contact angle increased from approximately 65.6° near 700 °C to 85.3° near 500 °C, while the droplet width decreased from approximately 3.002 to 2.580 mm. The coupled increase in angle and approximately 14% contraction of the projected footprint is consistent with contact-line depinning and recession from the initially broad, metastable configuration. The original low-angle branch was not recovered during reheating, demonstrating thermal path dependence rather than a reversible equilibrium response.

After the first-cycle relaxation, the second heating and cooling branches showed substantially closer agreement. Over 500–700 °C, their temperature-matched mean absolute contact-angle difference was approximately 1.5°, and the final cooling branch yielded a mean absolute contact-angle of 84.8 ± 1.5°. The apparent angle varied from approximately 83° near 700 °C to approximately 85°–86° near 500 °C. Thus, the high-angle state was retained during cooling within the same experiment, although the cycle supports a range near 80°–85° rather than an exact value of 80°. Because liquid volume was not deliberately increased or decreased at fixed temperature, these thermal-cycle observations should not be interpreted as formal advancing and receding contact-angle measurement.

The slight decrease in the contact angle that was observed at higher temperatures can be attributed to several temperature-dependent interfacial effects. As temperature increases, the liquid surface tension of the melt typically decreases. The viscosity also reduced, allowing the droplet to spread more readily and approach its stabilized apparent shape. Additionally, modest reductions in the solid–liquid interfacial energy or a change in interfacial structuring at elevated temperatures can further promote wetting. Collectively, these effects lead to a small but measurable decrease in contact angle with increasing temperature.

These findings indicate that the contact angle of molten FLiNaK on HOPG is not purely an instantaneous function of temperature but depends significantly on the initial geometry of the solid pellet and the time allowed for stabilization or measurement. The transient evolution observed with trapezoidal pellets, either decreasing from >100° or increasing from <50° toward about 80°, suggests that molten FLiNaK undergoes a relaxation process governed by interfacial energy minimization and spreading kinetics.

These results indicate that the apparent discrepancies observed in high-temperature wetting studies of molten salts may stem from kinetic limitations rather than fundamental differences in interfacial energetics.^9,14^ Numerous studies reported very different contact angle values for molten salts on graphite or carbon surfaces, which underlines the lack of a universal contact angle. For example, a review summarizing a sessile drop measurement of fluoride salts on graphite showed FLiNaK contact angles ranging from about 90° to 135° depending on salt composition and graphite grade.^9^ Another more recent comprehensive wettability study of LiCl–KCl and FLiNaK salts on various substrates and graphite showed FLiNaK contact angles from 79° to 83°, which are significantly lower than previous published values.^14^ This large scatter suggests that many of the differences across studies may arise not from true differences in interfacial energetics but instead from kinetic limitations, differences in surface preparation, the substrate grade or roughness, the dosing method of FLiNaK, hold duration, and measurement methodology. From a practical standpoint, the present study underscores two key considerations for reliably measuring sessile drops at high temperature: (1) the choice of initial pellet geometry and (2) insight into when the FLiNaK–HOPG system reaches a stable contact angle or the stability of the contact angle from a long-duration experiment.

### Geometry Sensitivity of Apparent Contact Angle

To further emphasize the importance of a regular salt pellet geometry on contact angle measurements, additional experiments were conducted using FLiNaK salt chunks with an approximately 1.5 mm diameter and varying heights. Chunks were used because of the difficulty of obtaining dies of such dimensions, and this is the shape that researchers will default to instead of making a regular shaped pellet. Tests were conducted under the first temperature profile, which was the ramp-to-800 °C profile. Because of the irregular shape of the salt chunks, the left and right contact angles measured for the solid-state FLiNaK ranged widely from 19° to 134°. As shown in Figure 5, the 1.5 mm salt chunk with a nearly square shape exhibited slightly higher contact angle values than the 3 mm pellets, but the chunk maintained the same overall trend of a near-constant contact angle with increasing temperature. Because the lateral dimension of the HOPG substrate is much larger than the droplet contacts radius (<3 mm), the edge effect is negligible, and the small variation in contact angle likely reflects minor differences in surface flatness, cleaving quality, or thermal expansion rather than intrinsic differences in surface chemistry.^5,26,27^

**Figure 5:**
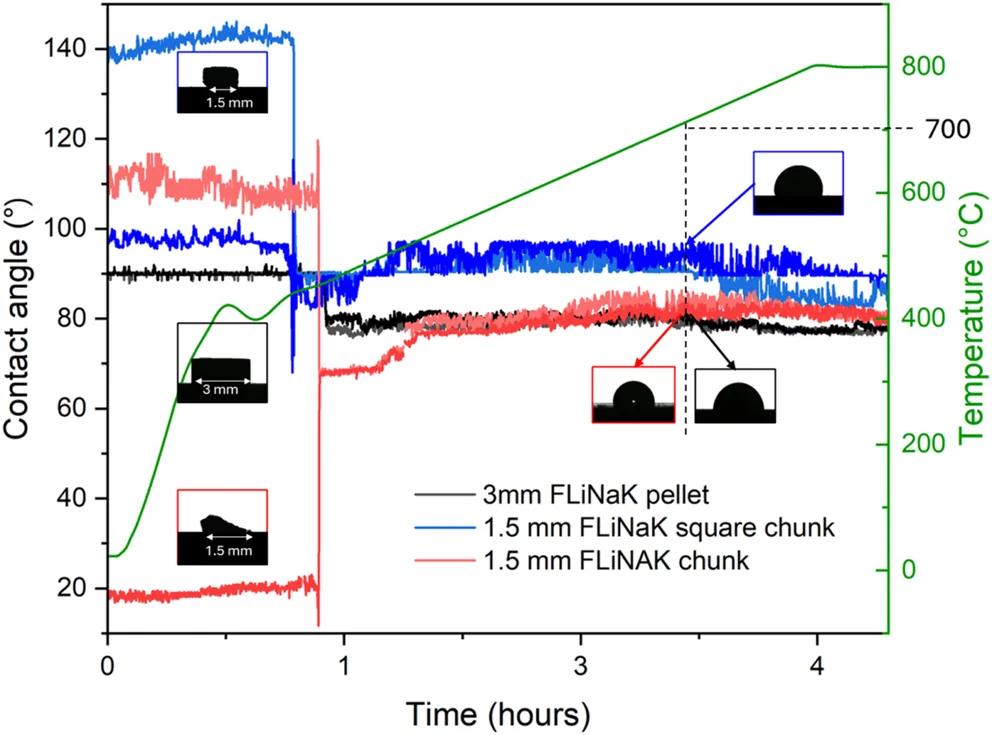
Apparent contact angle (dark color indicates left-side contact angle, and light color indicates right-side contact angle) of FLiNaK on HOPG as a function of temperature for pellets of different widths (1.5 mm FLiNaK chunk with different geometry and 3 mm FLiNaK pellet).

The relative importance of gravitational and surface tension forces during droplet spreading can be further evaluated by comparing the droplet size with the characteristic capillary length (*l*_c_). Capillary length represents the characteristic scale at which gravity begins to distort the shape of a droplet relative to the surface tension–dominated regime. Importantly, the capillary length is not a sharp threshold but rather a transition scale. When the droplet radius *R* ≪ *l*_c_, gravity has little influence, and the measured contact angle closely approximates the stabilized apparent contact angle. When *R* ≈ *l*_c_, both gravity and surface tension contribute, making the apparent contact angle sensitive to droplet size and initial geometry. For *R* ≫ *l*_c_, gravitational effects dominate, leading to significant distortion of the droplet profile and a geometry-dependent apparent contact angle. For FLiNaK, the capillary length^16^ is *l*_c_ = (γ_LG_/ρ*g*)^0.5^, where γ_LG_ is the surface tension of the salt, *g* is the acceleration due to gravity, and ρ is the density of the salt, which quantifies the length scale at which the capillary and hydrostatic pressure balance is close to 3 mm using ρ = 2164 kg/m^3^ and *g* = 9.8 m/s^2^. Table 1 shows the calculated capillary length at different temperatures with the surface tension of FLiNaK that is used for the calculation. Because the capillary length of molten droplets in this study was lower or at the threshold, both gravity and surface tension were expected to contribute in determining the droplet shape.

**Table 1:** Capillary Length and Bond Number of FLiNaK at Various Temperatures^a^

temperature (°C)	FLiNaK density (kg/m^3^)	FLiNaK surface tension (N/m)	capillary length (mm)	bond number for 3 mm pellet	bond number for 1.5 mm pellet
500	2165	0.194	3.024	0.246	0.062
600	2092	0.184	2.995	0.251	0.063
700	2019	0.174	2.963	0.256	0.064
800	1946	0.164	2.929	0.262	0.066

a Bond numbers were calculated in two cases of the pellet diameter.

The Bond number is a dimensionless parameter that quantifies the relative importance of gravitational force compared with surface tension. Thus, even if the capillary length is known, the Bond number should be evaluated because it incorporates the actual drop radius or pellet size used in the experiments. This allows us to quantify the degree of gravitational distortion and determine whether the measured contact angle is truly geometry-independent or biased by droplet size. The Bond number (Bo) is defined in eq ([Disp-formula eq1]) as^16^

1 $$\mathrm{Bo}=\frac{\Delta \rho gL^{2}}{\gamma_{\mathrm{LG}}}$$

where *L* is the droplet radius, and Δρ is the liquid–vapor density difference, which is approximated by the liquid density due to the low vapor pressure of FLiNaK.^17^ The FLiNaK density used in the Bond number calculation was evaluated from eq ([Disp-formula eq2])^28^

2 $$\rho \left(T\right)=2530-0.73\left(T+273.15\right)\left[\frac{\mathrm{kg}}{m^{3}}\right]$$

The surface tension of FLiNaK as a function of temperature (*T*, measured in degrees Celsius) was used based on eq ([Disp-formula eq3])^28^

3 $$\gamma_{\mathrm{LG}}\left(T\right)=272.6-0.1014\times \left(T+273.15\right)\left[\frac{\mathrm{mN}}{m}\right]$$

The Bond numbers at different temperatures (500, 600, 700, and 800 °C) with two different sizes, 3 and 1.5 mm, were calculated and are listed in Table 1. When Bo ≪ 1, surface tension dominates, and the droplet maintains a spherical-cap geometry with negligible gravitational distortion. Values approaching unity indicate that gravity and capillarity are comparable, leading to noticeable flattening and potential deviations in the apparent contact angle. For Bo ≫ 1, gravitational effects become dominant, and the droplet transitions toward puddle-like spreading. Because the Bond number scales with droplet size, the threshold at which gravity becomes significant corresponds to the capillary length, where Bo ≈ 1. This framework establishes a quantitative basis for interpreting wetting behavior, ensuring that observed contact angles reflect interfacial energetics rather than hydrostatic deformation.^18,19,29^

The Bond numbers approximately 0.06 for the 1.5 mm specimens and approximately 0.25 for the 3 mm specimens are both below unity, indicating that capillary forces dominate the overall droplet shape. However, the 3 mm case is not in the asymptotic B_0_ → 0 limit, and a modest contribution from gravity or size-dependent shape distortion cannot be excluded. Moreover, the 1.5 mm specimens were irregular chunks whereas the 3 mm specimens were pressed pellets. Therefore, initial geometry and droplet size were not independently varied. The observed differences are primarily consistent with geometry-dependent contact-line pinning and capillary relaxation, but a controlled same-geometry size series would be required to quantitatively decouple geometry, size and gravity.

### Surface and Interfacial Energy of FLiNaK–HOPG at High Temperatures

The stabilized apparent contact angle of FLiNaK on HOPG was observed to equilibrate around approximately 80° across the measured temperature range. Several analytical approaches exist for extracting solid surface energy from wetting data. Multiliquid component methods, such as Owens–Wendt^30^ or van Oss–Good–Chaudhury,^31,32^ are widely applied at ambient conditions because they can separate dispersive and polar contributions to the surface energy of the solid (γ_SG_). However, these require at least two chemically distinct probe liquids measured at the same temperature. At ambient conditions, this approach is routine and often preferred; however, it is effectively impractical in this study because no readily available, nonreactive molten salt liquids match the thermal conditions of FLiNaK on HOPG and graphite over the same applicable temperature range. In short, the experimental context forced a single-liquid inversion that immediately ruled out the standard two-liquid decomposition method as a realistic option for determining surface energy in this work.

Among single-liquid approaches, the Neumann EOS^20^ is the standard practical route because it provides a direct algebraic relation between the measurable contact angle, known liquid surface tension, and unknown solid surface free energy. The EOS avoids splitting surface energy into components and requires only the single-liquid probe that we measured earlier in this study and a numerical inversion. Practically, it is simple numerical root finding, and it allows us to exploit a temperature sweep of a single liquid to check the consistency and stability of the extracted surface energy across temperatures.

To convert the measured contact angle into an estimate of the HOPG surface free energy (γ_SG_), the Neumann EOS formulation combines Young’s relation with empirical EOS for the solid–liquid interfacial energy

4 $$\cos\theta =-1+2\sqrt{\frac{\gamma_{\mathrm{SG}}}{\gamma_{\mathrm{LG}}}}\exp\left[-\beta {\left(\gamma_{\mathrm{LG}}-\gamma_{\mathrm{SG}}\right)}^{2}\right]$$

Here, γ_LG_ represents the surface tension of FLiNaK at the give temperature, and β is an empirical interaction coefficient with units of meters squared per millijoules squared. The EOS approach requires only a single liquid probe whose surface tension is known at the specified temperature, making this method suitable for experimental work involving FLiNaK at high temperatures. The surface tension of FLiNaK as a function of temperature (*T*, measured in degrees Celsius) was used based on eq ([Disp-formula eq3]).^28^ The canonical value β = 1.247 × 10^–4^ m^2^/mJ^2^ was adapted following the original Neumann analyses.^20,21^

The surface energy of HOPG for the temperature range of 500 to 800 °C is illustrated in Figure 6. It shows the surface energy’s temperature dependence determined from FLiNaK contact angle measurements using the Neumann EOS approach. The blue markers represent the calculated surface energy values, and the gray error bars indicate the uncertainty arising from variations in the empirical parameter β, the FLiNaK surface tension, and the contact angle. The data reveals a monotonic decrease in surface energy with increasing temperature, which is consistent with the expected thermal weakening of intermolecular interactions at the HOPG surface.

**Figure 6:**
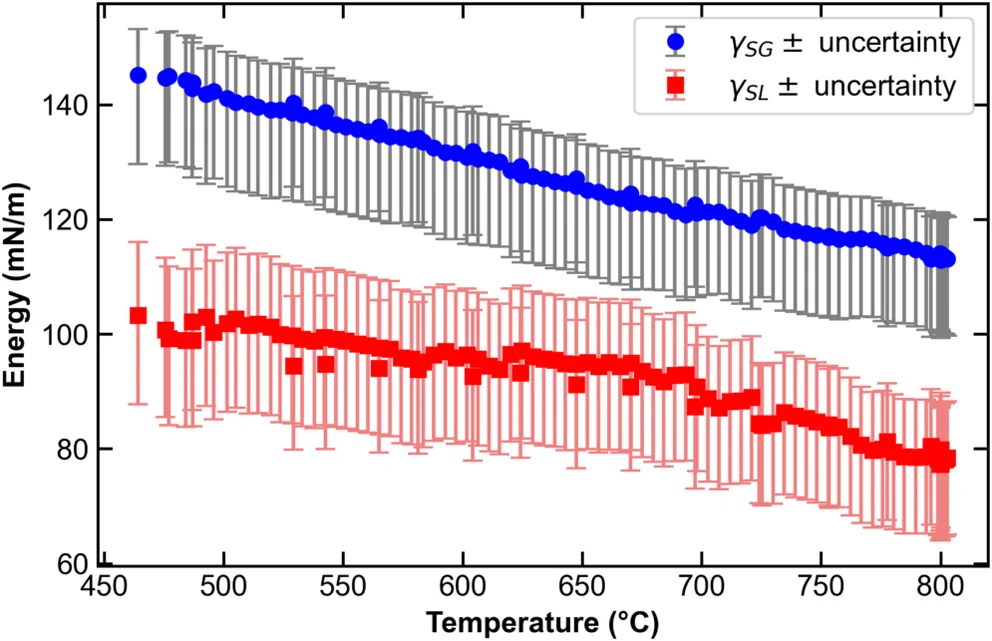
Temperature dependence of the apparent HOPG basal-plane solid–gas surface free energy γ_SG_ (blue circles), obtained from the Neumann EOS, and the FLiNaK-HOPG solid–liquid interfacial free energy, γ_SL_ (red squares), calculated from Young’s equation. Error bars show the sensitivity envelop associated with the stated variation β, θ, and γ_LG_. These bars do not represent the standard deviation between independent experimental replicates. Independent duplicate contact angle experiments yielded stabilized apparent angles within 2% and this observed reproducibility is encompassed by the ±2° contact angle variation included in the sensitivity analysis.

The interfacial energy between the solid and the liquid (γ_SL_) is a key thermodynamic quantity governing wetting, capillary infiltration, and interface stability. Once the apparent solid energy (γ_SV_) was determined from the Neumann EOS approach, γ_SL_ was back-calculated using Young’s eq (eq 5)­

5 $$\gamma_{\mathrm{SL}}=\gamma_{\mathrm{SG}}-\gamma_{\mathrm{LG}}\cos\theta$$

Using the calculated surface energy and contact angle, the resulting interfacial energy values (Figure 6) decrease gradually from approximately 100 mN/m at 500 °C to about 85 mN/m at 800 °C, showing a similar thermal trend to surface energy but with a consistently lower magnitude, as expected for a partially wetting system. The relatively modest temperature dependence of γ_SL_ indicates that the energetics of the FLiNaK–HOPG interface are largely stable across the investigated molten salt operating window, which is consistent with the minimal change in apparent contact angle with temperature.

The extracted interfacial energies (γ_SL_) in this study were consistently 30–40 mN/m lower than the corresponding solid surface energies (γ_SV_), reflecting the modest but finite wetting tendency of molten FLiNaK on HOPG. This magnitude of difference is consistent with the measured contact angle through Young’s relation and indicates that wetting is governed primarily by physical adsorption and van der Waals interactions rather than chemical bonding. In contrast, a system exhibiting reactive wetting typically shows a sharp reduction in γ_SL_ (often by 100–500 mN/m) and a corresponding decrease in contact angle owing to interfacial reactions, dissolution, or compound formation, which release interfacial energy and promote spreading.^33^ The absence of such a trend in the present results, combined with the nearly constant offset between surface energy and interfacial energy across 500–800 °C, suggests that the graphite basal-plane energetics remain thermally stable and chemically inert. From the perspective of our modeling results, surface corrugation reduces the effective contact area and weakens interactions with polar liquids. Therefore, these structural modifications provide a plausible mechanism for the experimentally observed slight slope changes in solid–liquid and solid–gas surface energies across the 700–800 °C temperature range.

To assess the robustness of these results, a sensitivity analysis was performed by varying key parameters within realistic uncertainty ranges: β by ±50%, θ by ±2°, and γ_LG_ by 2 mN/m. These variations produced changes in the calculated γ of less than ±5%–10% across the studied temperature range. The surface energy decreased systematically with temperature from approximately 145 mJ/m^2^ at 480 °C to 115 mJ/m^2^ at 800 °C. The relatively modest influence of parameter perturbations indicates that the observed invariance of γ, with temperature is a reliable outcome even though the absolute values are subjected to uncertainty. Across the measured temperature series, in which the contact angle of FLiNaK remained approximately 80°, the surface energy value of HOPG values likewise showed little temperature dependence.

It should be noted, however, that the EOS constant β was originally derived from data involving primarily organic liquids on polymeric and inorganic solids at ambient conditions. Therefore, its transferability to molten fluoride salt on HOPG at 500–800 °C has not been independently validated. Additionally, the inversion also treats the stabilized apparent contact angle as an approximation to the Young angle and therefore does not explicitly account for hysteresis, crystallographic anisotropy, surface heterogeneity, or possible temperature-dependent interfacial chemistry. In addition, a single-liquid EOS does not resolve separate dispersive and polar contributions. Varying β by ±50%, θ by ±2°, and γ_LG_ by 2 mN/m changed the calculated values by approximately 5–10% over the investigated range. The absolute γ_SG_ and the γ_SL_ values subsequently calculated from Young’s equation, should therefore be regarded as model-dependent semiempirical estimates. The relative temperature trend is more defensible than the absolute magnitude. Because γ_SL_ is calculated from γ_SG_ and the same θ and γ_LG_ inputs, the uncertainties in γ_SG_ and γ_SL_ are correlated rather than independent.

The magnitude of our results falls between the defect-free basal values obtained by inverse gas chromatography (∼63 ± 7 mJ/m^2^)^34^ and the upper-bound cleavage energy (∼195 mJ/m^2^).^35^ In previous studies, atomistic simulation predictions showed similar results (∼75 mJ/m^2^)^36^ and experiment-derived values using probe liquids at ambient conditions (∼62 mJ/m^2^).^37^ This placement is consistent with the interpretation that the EOS method yields an effective macroscopic surface energy, which accounts for ideal basal-plane contributions and possible finite-temperature, defect, and other interactions with a reactive, high-temperature molten salt environment. Importantly, although absolute magnitude is model-dependent due to the empirical EOS parameter β, the decreasing trend with temperature (Figure 6) shows meaningful insight into high-temperature wetting behavior. The discrepancy with low-temperature inverse gas chromatography (IGC), MD, and experiment-derived values underscores the importance of sensitivity analysis. In this context, our estimates supplement existing low-temperature values by offering the first high-temperature assessment of HOPG surface energy in a molten salt system. Taken together, the placement of our EOS-derived values between low-temperature, adsorption-based estimates and cleavage upper bounds, along with their systematic but moderate decrease with temperature, indicates that the energetics of the HOPG basal plane remain relatively stable even in the aggressive high temperatures of FLiNaK.

In the experiment/EOS analysis, γSG and γSL denote apparent interfacial free energies, i.e., free energy per unit area entering the Young/EOS description. In contrast, the MD slab-bulk calculation based on time-averaged total energies yields an excess surface energy. Because the entropic contribution is not evaluated separately, the MD quantity is not a rigorous finite-temperature surface free energy. The two quantities are therefore compared in magnitude and trend, but not treated as strictly identical.

Although the uncertainty analysis of the empirical parameter β indicates consistent trends in the estimated surface energy of HOPG, additional validation is desirable to further support the temperature-dependent behavior. Therefore, MD simulations were performed to provide complementary bounds and independently examine the surface energy variation with temperature.

To capture structural variability in realistic HOPG, two representative models were considered: (1) pristine graphene stacking and (2) polycrystalline graphene stacking along the *c*-axis, as shown in Figures 7(a) and S2. This dual-model approach enables evaluation of the influence of in-plane grain boundaries and stacking disorder on the effective surface energetics.

**Figure 7:**
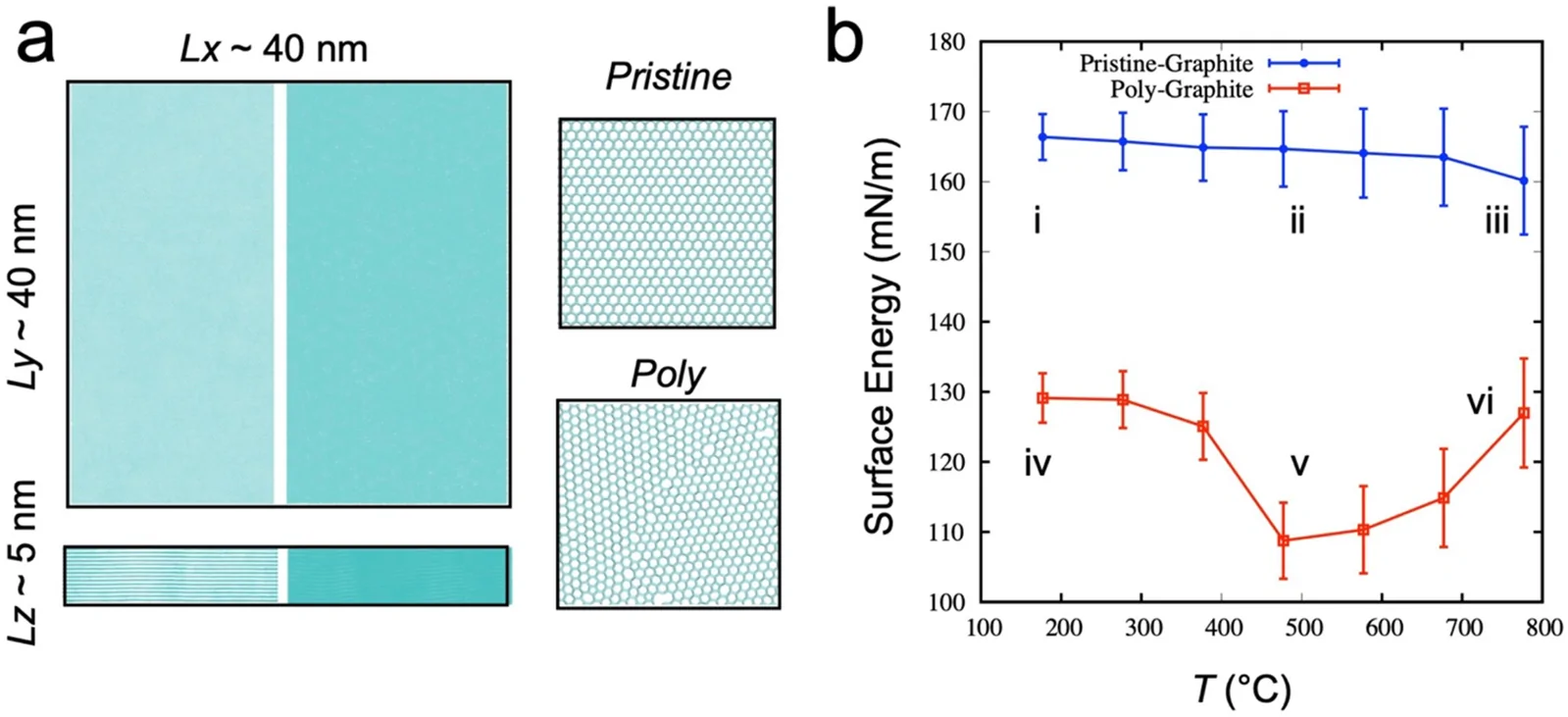
(a) Simulation models of HOPG graphite used for MD calculations. The systems have lateral dimensions of approximately 40 × 40 nm and a thickness of approximately 5 nm along the *c*-axis. Representative top-view structures of pristine graphite and polycrystalline graphite are shown; the polycrystalline model contains grain boundaries composed of characteristic 5–7 ring defects. (b) Calculated surface energy of the graphite (001) surface as a function of temperature for (blue) pristine and (red) polycrystalline models. Error bars represent statistical uncertainties obtained from time-averaged production simulations. The surface height distributions for (i)–(vi) are provided in Figure S3.

Intralayer carbon–carbon interactions were described using the AIREBO potential,^38,39^ and interlayer interactions were modeled with a Lennard–Jones potential to account for van der Waals cohesion between graphene sheets. Polycrystalline monolayers were generated using a previously reported construction algorithm,^40,41^ producing well-stitched grain boundaries with ring defects commonly observed experimentally.^23,24,42^ To prevent artificial registry locking and mimic realistic stacking disorder, individual graphene layers were randomly shifted in-plane before stacking.

Figure 7(b) presents atomistic modeling results and calculated surface energies. The surface energy of the graphite (001) surface was evaluated using eq ([Disp-formula eq6])

6 $$\gamma_{\mathrm{SV}}=\frac{E_{\mathrm{slab}}-E_{\mathrm{bulk}}}{2A}$$

where *A* denotes the in-plane surface area.

The pristine graphite model exhibits a monotonic decrease in surface energy from approximately 170 to 160 mN/m with increasing temperature. In contrast, the polycrystalline model yields substantially lower surface energies, decreasing from about 130 to 110 mN/m. This reduction of the polycrystalline model arises not only from enhanced out-of-plane deformation at grain boundaries but also from local stacking registry mismatch. Although pristine graphite preserves energetically favorable AB stacking, the polycrystalline model contains regions where this ideal registry is disrupted. Such a stacking disorder weakens interlayer van der Waals cohesion and thereby lowers the effective surface energy. Notably, the experimentally calculated surface energy of graphite (120–140 mN/m) lies between the pristine and polycrystalline predictions. This agreement supports the view that realistic HOPG consists of both well-ordered domains and defect-containing regions.

In the polycrystalline case, a change in slope is observed around 500 °C, suggesting temperature-induced surface rearrangement due to grain boundaries. To elucidate the structural origin of this behavior, we analyzed the surface morphology of both pristine and polycrystalline graphite models. Representative surface height maps are shown in Figure S2, in which the color scale indicates local height variation (blue corresponding to the highest reference level and red to −20 Å relative displacement).

Although the overall lateral dimensions remain nearly unchanged with increasing temperature, distinct differences emerge at the surface. The pristine graphite model retains a relatively flat morphology even at elevated temperatures. In contrast, the polycrystalline model undergoes noticeable surface reconstruction, exhibiting progressively enhanced out-of-plane deformation as temperature increases.

Notably, the average grain size in the present polycrystalline models (∼5 nm) is substantially smaller than that of typical HOPG samples, which exhibit microscale domains. Consequently, the degree of surface corrugation observed in our simulations likely represents an upper-bound scenario. In realistic HOPG, where large pristine domains dominate, such deformation effects can be partially suppressed.

The EOS-derived apparent HOPG basal-plane surface free energy decreases from approximately 145 mJ/m^2^ near 500 °C to approximately 120 mJ/m^2^ at 800 °C. The pristine-graphite MD values are higher, approximately 165–170 mJ/m^2^ at a lower temperature and approximately 160 mJ/m^2^ at 800 °C but show a similarly weak negative temperature slope. The polycrystalline model yields lower values, approximately 108–130 mJ/m^2^, which overlap the EOS magnitude. However, its temperature dependence is nonmonotonic, with a minimum near 500 °C followed by an increase toward 800 °C. Thus, the MD calculations bracket the order of magnitude of the EOS estimates but do not reproduce a single common temperature trend and should not be interpreted as a direct validation. The differences are consistent with the distinct quantities and idealization involved. The MD calculation evaluates an energetic slab-bulk surface excess for graphite in the absence of FLiNaK, while the EOS infers an apparent interfacial free-energy quantity from a semiempirical wetting model. The highly defect-rich approximately 5 nm grain model also represents an upper-bound disorder case rather than the microstructure of ZYA-grade of HOPG. Agreement in magnitude supports the plausibility of the inferred range, whereas disagreement in detailed temperature dependence highlights the model dependence and the need for future simulations that include the molten-salt interface and free-energy contribution.

The combined observation of shape-dependent droplet behavior and the nearly temperature-invariant surface energy of HOPG in contact with FLiNaK has important implications for high-temperature graphite applications. First, the results demonstrate that experimental protocols must carefully control initial droplet geometry to ensure reproducible wettability data because apparent variations in θ may arise from the kinetic shape effect rather than intrinsic changes in surface chemistry. Second, the surface energy values obtained by the EOS method suggest that the basal-plane energetics of graphite are stable across the relevant molten salt operating window.

This stability provides a reliable reference point for evaluating more complex nuclear-grade graphite materials, where porosity, crystallite orientation, and impurity chemistry can introduce additional variability in salt infiltration and wetting behavior. Thus, the present study establishes a baseline methodology for extracting γ_s_ from high-temperature sessile drop experiments and provides essential context for interpreting graphite–salt interactions in MSR environments.

## Conclusion

This study investigated the wetting behavior of molten FLiNaK on HOPG across a temperature range of 500–800 °C. Sessile drop experiments revealed that apparent contact angles were influenced by the initial droplet geometry, underscoring the need for careful control of pellet preparation and measurement protocols when assessing high-temperature wetting behavior.

By applying the Neumann EOS, we extracted surface energy values for HOPG from the contact angle and surface tension data. The results showed only a modest decrease in surface energy (γ_s_) with temperature, with values spanning approximately 145 mN/m at 500 °C to 120 mN/m at 800 °C. Sensitivity analysis confirmed that the observed trend is robust within experimental uncertainty, although the absolute values remain semiempirical due to the extrapolation of the EOS interaction parameter β beyond its original ambient temperature derivation.

These findings provide two key insights. First, they establish a reproducible methodology for extracting solid surface energies in molten salt environments using single-liquid data—a necessity given the impracticality of multiliquid methods at high temperatures. Second, they provide a reliable baseline for the HOPG basal plane against which the more complex wetting behavior of nuclear-grade graphite can be evaluated thoroughly. Because the performance of graphitic materials in MSRs depends on their resistance to salt infiltration and interfacial degradation, a clear understanding of the intrinsic surface energetics of HOPG is an essential step toward interpreting and predicting the behavior of practical graphite components. Future work should extend this analysis to polycrystalline nuclear-grade graphite materials and evaluate the role of surface chemistry and irradiation damage on salt wetting and infiltration.

## Supplementary Material



## Notes

Thermal cycle of the apparent contact angle and projected width of FLiNaK on HOPG, stabilized apparent contact angle from independent duplicate experiments, and MD Supporting Information (detailed model description and surface height distributions) (PDF)

## Abbreviations

EOS: equation of state
FLiNaK: LiF–NaF–KF
HOPG: highly oriented pyrolytic graphite
MSR: molten salt reactor.
