# Supplementary material for: High-Temperature Surface Energy and Interfacial Energy of the Molten FLiNaK–Highly Oriented Pyrolytic Graphite (HOPG) System from Selected Contact Angle Measurement
Source: Ind Eng Chem Res. 2026 Sep 18;65(38):20432–42. doi: 10.1021/acs.iecr.6c02718 (PMC13632433; doi:10.1021/acs.iecr.6c02718)
Supplement: Supplementary file 1 [file ie6c02718_si_001.pdf]

# Supporting Information

# High-temperature surface energy and interfacial energy of molten FLiNaK–highly oriented pyrolytic graphite (HOPG) system from selected contact angle measurement

*Jisue M. Braatz[1*], Gang Seob Jung[2], Nidia C. Gallego[3]*

[1] Radioisotope Science Technology Division, Oak Ridge National Laboratory, 1 Bethel Vally Rd, Oak Ridge, TN 37830, United States, moonj1@ornl.gov

[2] Computational Sciences and Engineering Division, Oak Ridge National Laboratory, Oak Ridge, TN 37831, USA

[3] Chemical Science Division, Oak Ridge National Laboratory, 1 Bethel Vally Rd, Oak Ridge, TN 37830, United States

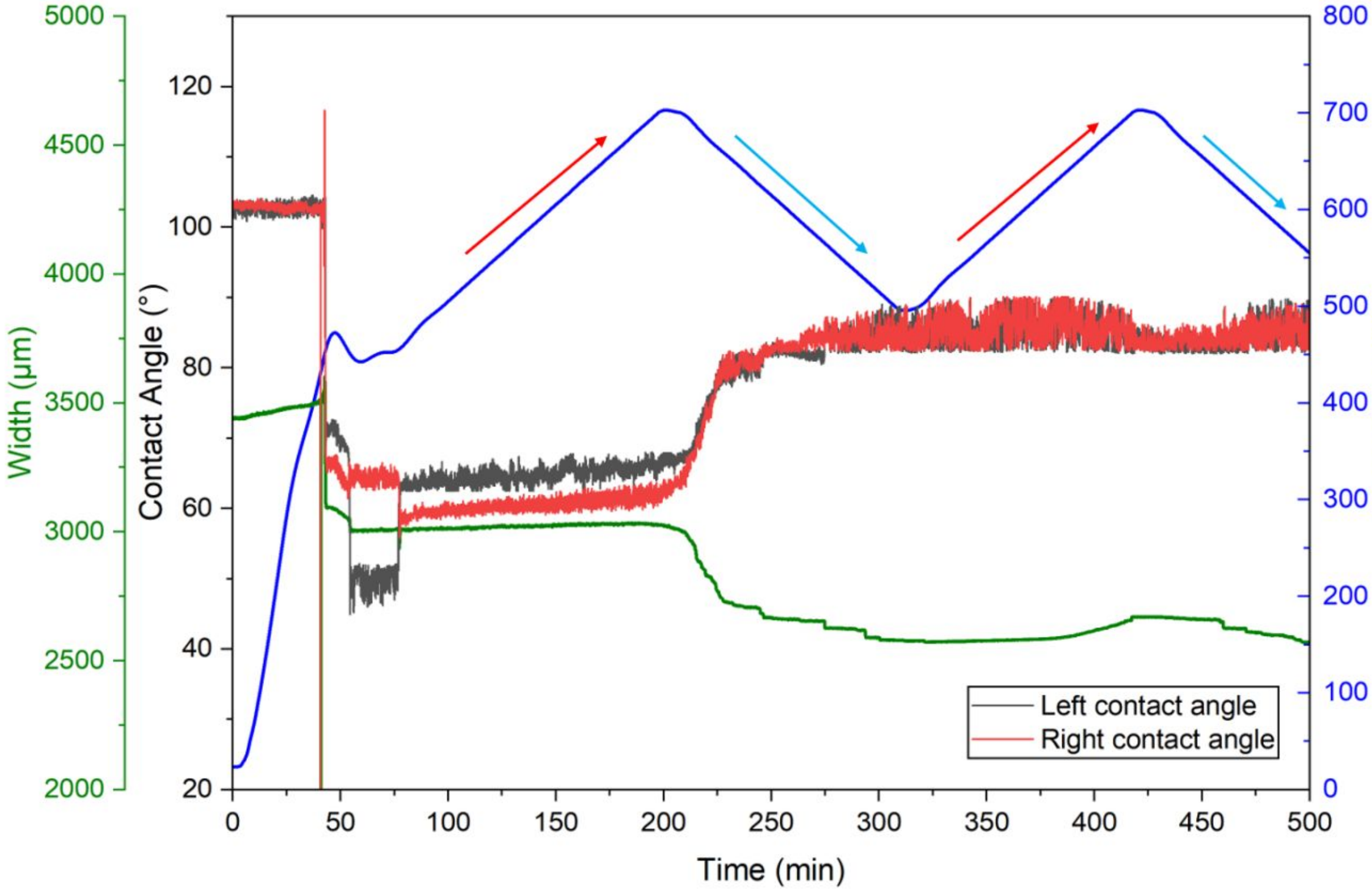


**Figure S1**. Thermal-cycle dependence of the apparent contact angle(black, red) and projected droplet width(green) for molten FLiNaK on HOPG. The apparent angle is the left- and right-side measurements. Arrows indicate first heating, first cooling, second heating, and final cooling between approximately 500 and 700 °C. The first cooling branch shows a transition from broad, low angel configuration to a narrower, high-angle configuration, whereas the subsequent heating and cooling branches show substantially smaller path dependence. Data below the FLiNaK melting region is not interpreted as liquid contact angles.

**Table S1.** Stabilized apparent contact angles from independent duplicate experiments. Each run value represents the mean of the left- and right-contact angle over the defined stabilized interval. RPD denotes relative percent difference between independent runs.

| Pellet configuration | Run 1 stabilized angle | Run 2 stabilized angle | Absolute difference | RPD[1] |
|---|---|---|---|---|
| Straight edge | 77.5° | 79.8° | 2.3 | 2.92% |
| Wide trapezoid side down | 77.5° | 78.9° | 1.4 | 1.79% |
| Narrow trapezoid state down | 81.0° | 82.2° | 1.2 | 1.47% |

[1] Relative Percent Difference (RPD) = $\frac{|\theta_1-\theta_2|}{(\theta_1+\theta_2)/2} \times 100$

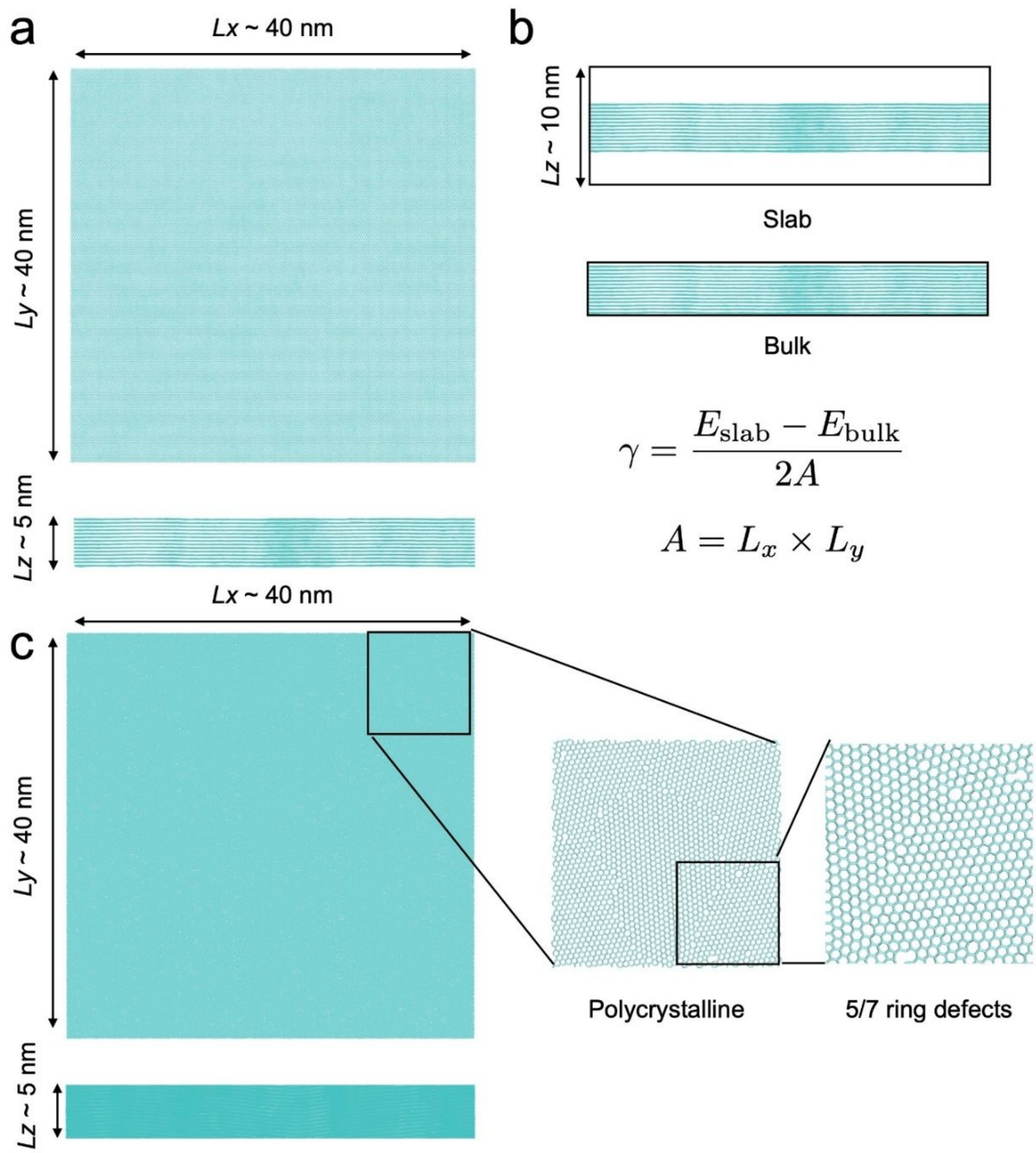


**Figure S2. Molecular dynamics models for HOPG surface energy calculation.** (a) Simulation cell geometry for pristine graphene stacking used to represent ideal HOPG. (b) Schematic illustration of the slab and bulk configurations used to compute the surface energy. (c) Polycrystalline graphene stacking model constructed using polycrystalline graphene monolayer. The in-plane structure consists of randomly oriented grains connected by well-stitched boundaries characterized by 5–7 ring defect motifs. A magnified view highlights the grain structure and representative 5-7 ring defects, which are commonly observed in experimental graphene systems. The stacked model along the c-axis is used to assess the influence of in-plane disorder on the effective surface energetics.

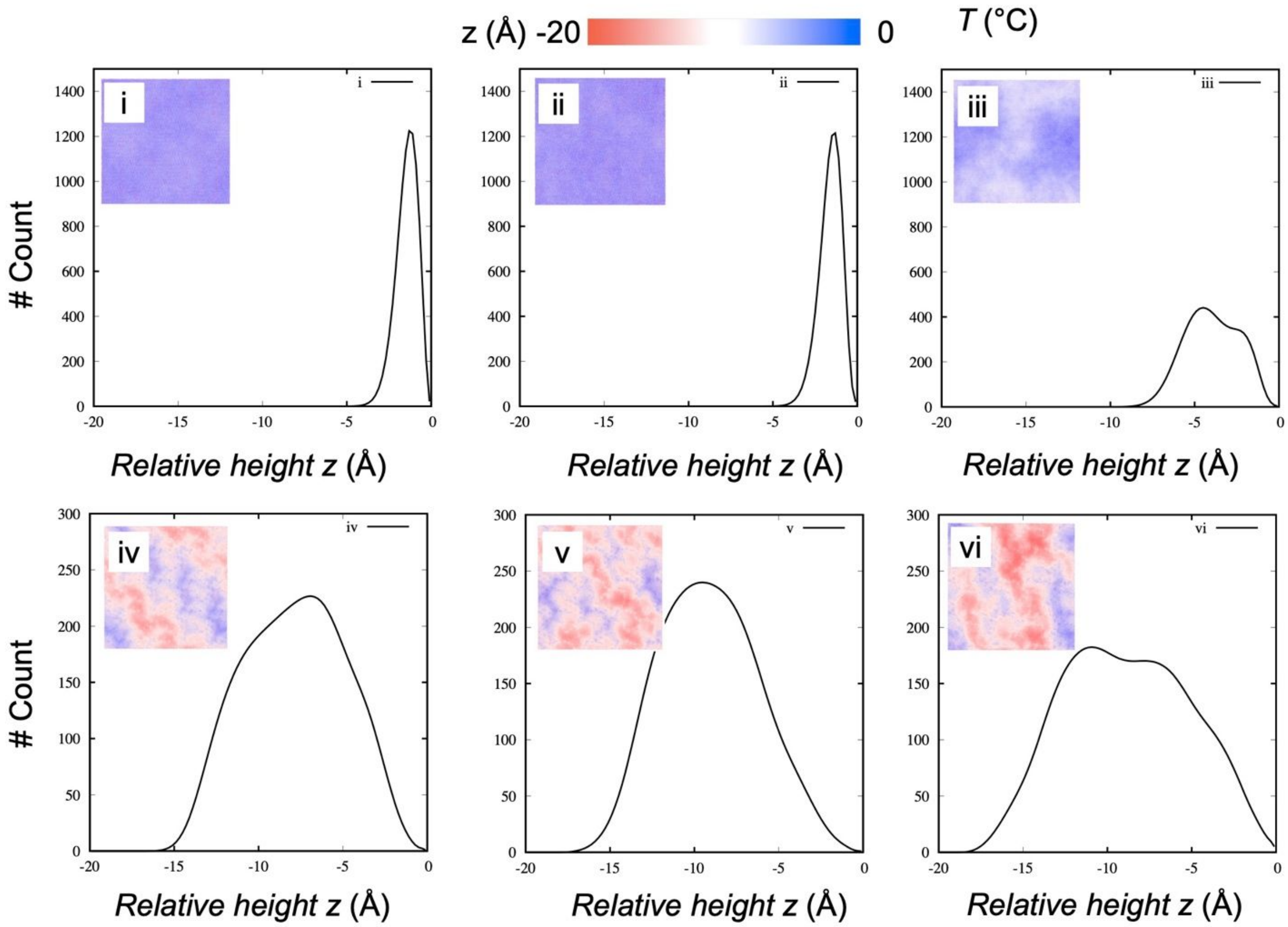


**Figure S3**. Surface height distributions correspond to the configurations labeled in Figure 6 panel (b). Insets show representative surface height maps, while the curves represent histograms of the relative surface height z. The color scale indicates relative height (Å), where blue denotes the highest reference level and red corresponds to -20 Å. Compared with pristine graphite (i–iii), the polycrystalline surfaces (iv–vi) exhibit broader height distributions and more pronounced out-of-plane corrugation at elevated temperatures.
